# Extraction, Radical Scavenging Activities, and Chemical Composition Identification of Flavonoids from Sunflower (*Helianthus annuus* L.) Receptacles

**DOI:** 10.3390/molecules26020403

**Published:** 2021-01-14

**Authors:** Zian Qiao, Lu Han, Xinsheng Liu, Huining Dai, Changmin Liu, Min Yan, Wannan Li, Weiwei Han, Xinlu Li, Silu Huang, Bo Gao

**Affiliations:** 1 School of Life Sciences, Jilin University, Changchun 130012, China; qiaoza18@mails.jlu.edu.cn (Z.Q.); luhan@jlu.edu.cn (L.H.); liuxs18@mails.jlu.edu.cn (X.L.); daihn18@mails.jlu.edu.cn (H.D.); liucz19@mails.jlu.edu.cn (C.L.); yanmin19@mails.jlu.edu.cn (M.Y.); liwannan@jlu.edu.cn (W.L.); weiweihan@jlu.edu.cn (W.H.); xinlu19@mails.jlu.edu.cn (X.L.); slhuang18@mails.jlu.edu.cn (S.H.); 2 Third-Level Laboratory of National Administration of Traditional Chinese Medicine, Jilin University, Changchun 130012, China; 3 Key Laboratory for Evolution of Past Life and Environment in Northeast Asia, Jilin University, Ministry of Education, Changchun 130012, China; 4 Key Laboratory for Molecular Enzymology and Engineering, Jilin University, Ministry of Education, Changchun 130012, China

**Keywords:** sunflower (*Helianthus annuus* L.), flavonoids, response surface methodology, radical scavenging activities, chemical composition

## Abstract

This study was focused on extraction, radical scavenging activities, and chemical composition identification of total flavonoids in sunflower (*Helianthus annuus* L.) receptacles (TFSR). We investigated the optimal extract parameters of TFSR using response surface methodology. The highest yield of TFSR was 1.04% with the ethanol concentration 58%, the material-to-liquid ratio 1:20 (*v*/*w*), the extraction time 2.6 h, and the extraction temperature 67 °C. The results of radical scavenging activities showed that ethyl acetate fraction (EAF) was the strongest by using 2-diphenyl-1-picrylhydrazyl (DPPH), 2, 2’-azino-bis (3-ethylbenzo thiazoline-6-sulfonic acid) (ABTS) and iron ion reducing analysis. The EAF had the highest flavonoids contents. Four fractions A, B, C and D were enrichment from EAF by polyamide resin. Fraction B had the highest flavonoids content. Thirteen chemical components of flavonoids in fraction B were first identified by Ultimate 3000 Nano LC System coupled to a Q Exactive HF benchtop Orbitrap mass spectrometer (UHPLC-HRMS/MS). Among of the thirteen chemical components, isoquercetin and daidzein were identified accurately by comparing with standard samples. Radical scavenging analysis showed that isoquercetin and EAF had strong activities. Therefore, sunflower receptacles can be used as a source of natural flavonoids. TFSR as a natural radical scavenger has potential applications in pharmaceutical industry.

## 1. Introduction

Sunflower (*Helianthus annuus* L.) is a species of Compositae, which has varieties of nutritional functions and medicinal benefits [1]. Sunflower is commercially grown all over the world, especially has the higher production in China, Argentina, Russia and France. At present, the research on sunflower mainly focused on seeds [1,2,3,4]. However, sunflower receptacles have not been researched as popularly as seeds.

Some sunflower receptacles were used as feed. Most of sunflower receptacles were burned as “waste” in field, which caused serious environmental pollution. Therefore, utilization of these “waste” can reduce environmental pollution [5]. In order to develop new functions of sunflower receptacles, some studies were conducted on its medicinal value. In Chinese folk, sunflower receptacles were used as a main part of the medicines, sunflower receptacles had a therapeutic function on hypertension, headache, dizziness, tinnitus, abdominal pain, dysmenorrhea, and uterine bleeding in the Grand Dictionary of Chinese Medicine (the second edition, 2012). So sunflower receptacles may have some active ingredients. However, the effective medicinal compounds of sunflower receptacles were still unclear, which limited its application in pharmaceutical industry.

In natural compounds, flavonoids had attracted much attention because of their specific biological activities. Flavonoid is a kind of natural compounds with a 2-phenylchromone structure and it is an important secondary class metabolite with low molecular weight that is found in plants [6]. Many flavonoids have antioxidant function and medicinal value, such as ipriflavone, which was reported had the potential neuroprotective effect, the neuroprotective effect of ipriflavone was related to its anti-inflammatory and antioxidant activities. Many medicinal mechanisms of flavonoids were related closely to radical scavenging activity [7].

In previous studies, four flavonoids were identified from flowers of sunflower, including hymenoxin, sudaehitin, hispidulin and pectolinarigenin [8,9]. Six flavonoids were identified from leaves of sunflower, including demethoxysudachitin, acerosin, sideritiflavone, xanthomicrol, methoxysudachitin, and nevadensin [10]. The identification of flavonoids in sunflower receptacles have not been reported until now. The chemical compounds of sunflower receptacles may be the material basis of its biological activities, so it is meaningful to identify the chemical composition of sunflower receptacles.

The aim of this research was to obtain the flavonoids from sunflower receptacles, assay their potential as antioxidants and the chemical composition. This research provided reliable data for studying flavonoids of sunflower receptacles in pharmaceutical industry.

## 2. Materials and Methods

### 2.1. Plant Materials and Chemicals

Sunflower receptacles were collected from Da’an City, Jilin Province (123°12′45″ E, 44°52′23″ N) in October 2019 and identified by professor Shuwen Guan of the School of Life Sciences, Jilin University. Isoquercetin standard sample, daidzein standard sample, rutin standard sample, butylated hydroxytoluene (BHT) were purchased from Meilun Biology Co. (Dalian, Liaoning Province, China). Iron ion reduction capacity kit was purchased from Congyi Biology Co. (Shanghai, China). NaNO_2_, AlCl_3_, NaOH, ABTS and DPPH were purchased from Aladdin Co. (Shanghai, China). Methanol and formic acid (Chromatographic grade purity) were purchased from Beijing Reagent Co. (Beijing, China). The ethanol, petroleum ether, ethyl acetate, n-butanol (analytical reagent grade) were purchased from Beijing Reagent Co. (Beijing, China).

### 2.2. Content Determination of TFSR

The contents of flavonoids were determined by aluminum nitrate colorimetry using rutin as standard [11]. A standard solution was prepared using a rutin solution (0–60 μg/mL). Each extract (1 mL) was mixed with NaNO_2_ (0.3 mL, 5%). After 6 min, AlCl_3_ (0.3 mL, 10%) and NaOH (2 mL, 1 M/L) were added to the mixture. Finally, the mixture was adjusted to 10 mL with 30% ethanol. After 15 min, the absorbance was read at 510 nm. The extraction yield of TFSR was listed as mg of rutin equivalent per gram of sunflower receptacles powder. The contents of TFSR were presented in milligram rutin equivalent per gram of dry weight extract (mg RTE/g DW) [12].

### 2.3. Response Surface Optimization of Extraction Conditions

Response surface method (RSM) was first proposed by Box and Wilson in 1951 [13]. Response surface was a commonly used method in flavonoids extraction optimization [14]. RSM is an experimental optimization technique based on experimental simulation to achieve the best response value. Different factors influence each other in the extraction experiment. RSM accurately assesses the correlation between these factors [15,16].

Preliminary experiments showed that extraction time, ethanol concentration, material-to-liquid ratio, and extraction temperature had a significant impact on the extraction yield. Improving the extraction yield was important to analyze TFSR. Therefore, the Box–Behnken design provided by Design Expert 11 (Stat Ease Inc., Minneapolis, MN, USA) was used. In this study, the independent variables were extraction time, ethanol concentration, material-to-liquid ratio and extraction temperature. The dependent variable (Y) was the extraction yield of TFSR. The optimal extraction process conditions for TFSR extraction were explored. The whole design consisted of 29 experiments, including 24 analysis factors and 5 central experiments [17]. The design of experiment is shown in Table 1.

### 2.4. Preparation of the Crude Extract and Fractions

According to the previously described protocol with some modifications [18,19,20], the whole sunflower receptacles were ground into fine powder (1.5 kg) and extracted with the optimal extraction conditions. All filtrates were collected and dried to obtain crude extracts of 186 g. One hundred and fifty of gram of the crude extracts were suspended into water, three solvents (petroleum ether, ethyl acetate, n-butanol) with increasing polarities were successively used to partition plant extracts, the partitioning of each solvent was operated three times, the extract liquor were collected and concentrated by vacuum distillation at 60 °C, 0.8 MPa for 3–4 h. The remaining water was removed by using freeze dryer (Alpha 1-2 LD plus, Christ, Germany) with −52 °C, 0.052 mbar for 72 h. The dry fractions were obtained and recorded as petroleum ether fraction (PEF), ethyl acetate fraction (EAF), n-butanol fraction (nBUF) and water fraction (WAF). Then rutin was used as the standard to measure the flavonoids contents of dry fractions. All fractions were stored in airtight containers in the dark at −20 °C before use.

### 2.5. Radical Scavenging/Reducing Assays

In recent years, many methods had been used to estimate the radical scavenging capacity of natural products. The commonly assays involved DPPH, ABTS, iron ion reducing ability, hydroxyl radicals, bleaching of β-carotene, cupric ion reducing antioxidant capacity (CUPRAC), metal chelation, protection against cellular oxidative damage and so on [21]. In this research, the former three methods were selected for rapid assessment of radical scavenging antioxidant potential.

#### 2.5.1. DPPH Radical Scavenging Capacity Assays

DPPH scavenging assay was useful to evaluate the antioxidant content of crude fraction and their comparison with reference standard samples, being based on the hydrogen atom transfer reduction of the persistent DPPH radical [21,22,23]. The samples of extracts from sunflower receptacles with different concentrations (The final concentration were 625, 312.5, 156.25, 78.13, 39.06, 19.53, 9.77, 4.88, 2.44, 1.22, 0.61, 0.31 ug/mL, 0.5 mL each) and 0.08 mmol/L freshly prepared DPPH (3 mL each) were mixed in a test tube for 0.5 h incubation at room temperature in the dark. Then, absorbance at 517 nm was recorded. All measurements were made in triplicate (*n* = 3). Using Equation (1):

(1) $$\mathrm{DPPH}\;\mathrm{radical}\;\mathrm{scavenging}\;\mathrm{activity}\;\left(\%\right)=\frac{A_{0}-A_{c}}{A_{0}}\times 100$$

where *A*_0_ is the absorbance of DPPH radical solution and methanol solution. *A_c_* is the absorbance of DPPH radical solution with sample solution.

#### 2.5.2. ABTS Radical Scavenging Capacity Assay

For the ABTS assay, the method in reference [24,25] was adopted with some modifications. The stock solutions included 2.6 mM potassium persulfate solution and 7.4 mM ABTS solution. The working solution was prepared by mixing the two stock solutions in equal quantities and allowing them to react for 12 h at room temperature in the dark. Then, the solution was diluted by mixing 1 mL of ABTS solution with 42 mL of methanol to obtain an absorbance of 0.777 units at 734 nm. The ABTS solution was newly prepared for each experiment. The extracts of sunflower receptacles with different concentrations (The final concentration were 500, 250, 125, 62.5, 31.25, 15.63, 7.81, 3.91, 1.95, 0.97, 0.48, 0.24 ug/mL, 0.5 mL each) were allowed to react with 2 mL of the ABTS solution. After 6 min, the absorbance at 734 nm was recorded. The ABTS scavenging capacity of the extracts were compared with BHT (0.5 mL each), and percentage inhibition was calculated as the ABTS radical scavenging activity. All measurements were made in triplicate (*n* = 3). Using Equation (2):

(2) $$\mathrm{ABTS}\;\mathrm{radical}\;\mathrm{scavenging}\;\mathrm{activity}\;\left(\%\right)=\frac{{\mathrm{ABTS}}_{\mathrm{control}}-{\mathrm{ABTS}}_{\mathrm{sample}}}{{\mathrm{ABTS}}_{\mathrm{control}}}\times 100$$

where ABTS_control_ represents the absorbance without sample. ABTS_sample_ represents the absorbance with samples. BHT was used as the reference for comparison.

#### 2.5.3. Iron Ion Reducing Ability Assay

The determination reduction were based on the amount of Fe_4_(Fe[CN]_6_) produced as an indicator. The antioxidant can reduce potassium ferricyanide, and the ferrous ions were used to produce Prussian-blue. Fe_4_ (Fe [CN]_6_) has maximum absorption peak at 700 nm. A greater absorbance value indicates that a sample has stronger reducing power. Iron ion reducing ability was measured using kit according to the manufacturer’s instructions.

### 2.6. Preliminary Purification of EAF by Polyamide Resin

Polyamide resin has a good enrichment effect on flavonoids. Thus, polyamide resin was selected to further enrich flavonoids from EAF [26,27,28,29]. The polyamide resin was pretreated and added to the chromatography column (glass column diameter, 3.2 cm; height, 40 cm; filling height, 25 cm). EAF sample (2 g) was dissolved in 10 mL of ethanol, mixed with 10 g polyamide resin, and dried in a blast dryer at 60 °C. The dried mixture was spread flat on top of the polyamide column. The elution solvent was a water–ethanol system (0%, 30%, 70%, and 95% ethanol). The elution volume of each ethanol concentration were 3BV (column volume), the elution speed was 1 BV/h, and the elution time were 3 h. The eluate was concentrated by vacuum distillation at 60 °C, 0.8 MPa for 3–4 h. The freeze dryer was used for further drying (the freeze-drying parameters: −52 °C, 0.052 mbar), after 36 h, the dry material was obtained and marked as A, B, C, D. The contents of flavonoids in A, B, C, D were measured.

### 2.7. Qualitative Analysis

UHPLC-HRMS/MS has proved to be an important and powerful tool for identification of compounds. Q Exactive mass spectrometer is particularly suitable for screening compounds of non-target or target, it can also achieve a wide range of qualitative and quantitative applications. It can be widely used in compounds identification. The resolution was up to 140,000 FWHM, which can eliminate the interference of isobaric and isomeric compounds and improve the reliability of results when analyzing samples in complex matrices. For example, fenugreek is a famous medicinal plant used to treat diabetes, the flavonoids and metabolomics of fenugreek were analyzed by UHPLC-HRMS/MS [30,31].

#### 2.7.1. Chromatographic Conditions

RP-C18 column (150 × 210 mm, 1.8 μm; Welch) was used in this study. The mobile phases were 0.1% formic acid aqueous solution (A) and 0.1% formic acid acetonitrile (B). The gradient elution was performed as follows: 0–5 min, 98% A; 5–10 min, 98% A → 50% A; 10–15 min, 50% A → 20% A; 15–20 min, 20% A → 5% A; 20–25 min, 5% A → 5% A; 25–30 min and 5% A → 98% A. The flow rate was 0.3 mL/min. The autosampler temperature was 10.0 °C, the column temperature was 35 °C. Injection needle height was 2.00 mm, and injection volume was 10 µL [32,33].

#### 2.7.2. Mass Spectrometry Conditions

Ion source was electrospray ionization source (ESI) under positive and negative ion switching scanning mode. Scanning range was *m*/*z* 150–2000. The detection method was full mass scan with data-dependent tandem mass spectrometry (dd-MS2). The resolution was 70,000 in full mass scan and 17,500 in dd-MS2. The ion spray needle voltage was 3.8 kV (positive). The capillary temperature was 300 °C. The collision gas was high-purity argon (purity ≥ 99.999%). The sheath gases were nitrogen (purity ≥ 99.999%) and 40 Arb, and the auxiliary gas was nitrogen (purity ≥ 99.999%; heater temperature was 350 °C. Data acquisition time was 30.0 min.

### 2.8. Statistical Analysis

All experiments were carried out at least three parallel tests. One-way analysis of variance (ANOVA) and mean comparisons were performed on the contents of flavonoid using SPSS 20.0 (IBM Co., New York, NY, USA). The radical scavenging activities analysis performed with Graphpad Prism v.8.1 (GraphPad Software Inc., San Diego, CA, USA). The data of compounds were collected by the UHPLC-HRMS/MS system through CD2.1 (Thermo Fisher, Shanghai, China), and then the data were searched and compared with the datebase of mzCloud, mzVault and ChemSpider. The structure of compounds were drawn by Indraw V3.5 (Integle Co., Shanghai, China). The chromatogram and mass spectra of isoquercetin and daidzein were provided by the software Xcilabur 3.0 (Thermo Fisher, Shanghai, China).

## 3. Results and Discussion

### 3.1. Response Surface Optimization

#### 3.1.1. Statistical Analysis and Model Fitting

Determination of extraction yield according to the standard curve Equation (3). The experimental data of RSM were exhibited in Table 2. Through quadratic polynomial regression fitting, the regression Equation (4) of RSM for the extraction yield of flavonoids was obtained. The experimental model showed statistical extremely significance (*p* < 0.0001). The lack of fit can explain the degree of fitting between the experiments and model. the lack of fit was not significant; thus, the further optimization experiments were not required. The total decisive coefficient (*R*^2^) of the model was 0.9876, which indicated that the model could resolve the response value changes of 98.76%. The results showed that the model fitted very well. The correction coefficient of determination *R*^2^_Adj_ = 0.9752 indicated that the changes of 97.52% in the response value model came from the selected variables. The coefficient of variation of the response value *Y* (extraction yield) was 5.93%, which was lesser than 10%. Therefore, the experiment had good repeatability.

(3) $$y=94.915x-1.1646\left(R^{2}=0.999\right)$$

(4) $$\begin{array}{c} Y=1.040+0.1027A-0.1041B+0.0257C+0.0429D+0.0741AB-0.0021AC-0.0031AD-0.0186BC\\ -0.0845BD-0.0503CD-0.0843A^{2}-0.4583B^{2}-0.1424C^{2}-0.1922D^{2} \end{array}$$

According to the F value, the influence of the four factors on the extraction yield of flavonoids was B (ethanol concentration, %) > A (extraction time, h) > D (extraction temperature, °C) > C (material to liquid ratio, (*v*/*w*). The linear terms A and B, the interaction term CD and the quadratic terms A^2^, B^2^, C^2^, and D^2^ in the model showed extremely significant effects (*p* < 0.001). The linear term D, the interaction terms AB showed highly significant effects (*p* < 0.01). The linear term C and the interaction term CD had significant effect (*p* < 0.05).

#### 3.1.2. Interaction Analysis

**Figure 1:**
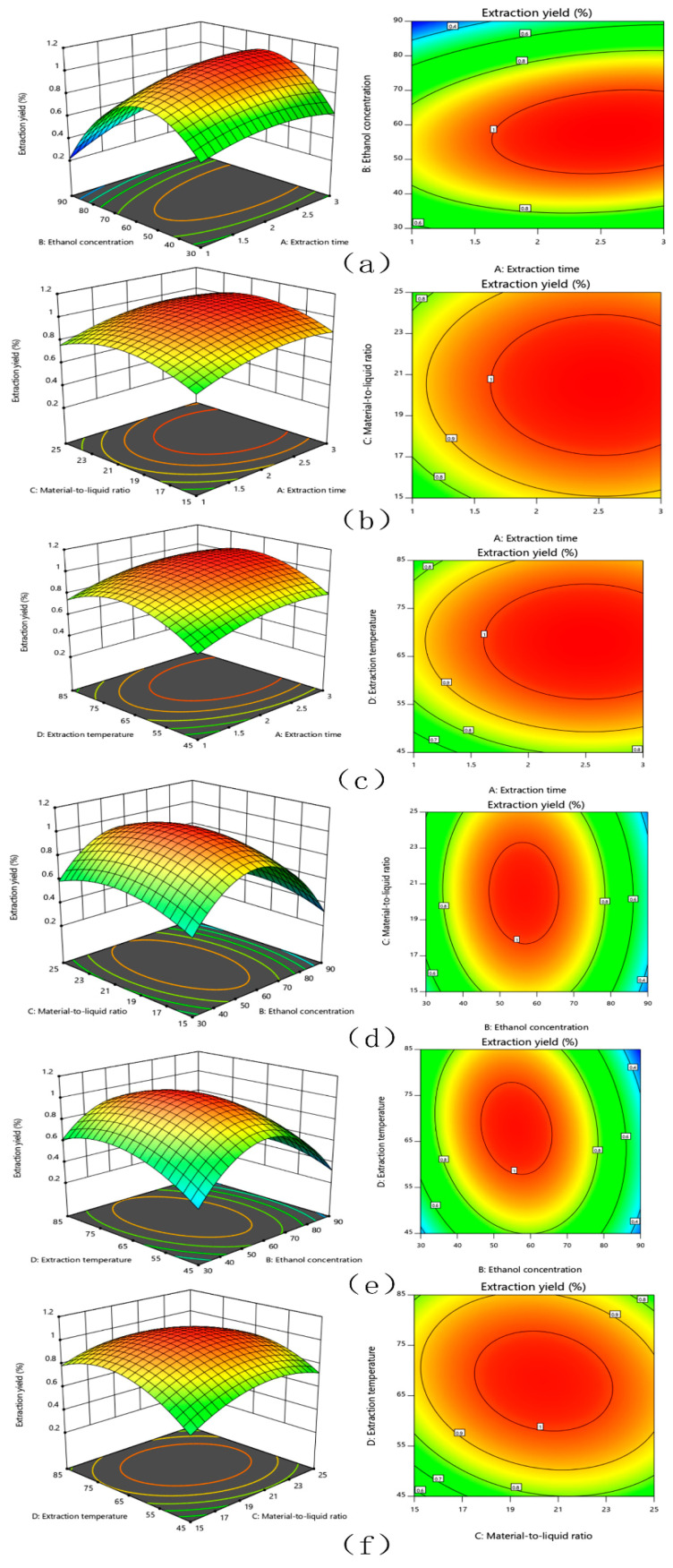
The mutual effects of response surface plots and contour plots for the extract yield of TFSR. (**a**) extraction time and ethanol concentration, (**b**) extraction time and material to liquid ratio, (**c**) reaction time and extraction temperature, (**d**) ethanol concentration and material to liquid ratio, (**e**) ethanol concentration and extraction temperature, (**f**) material to liquid ratio and extraction temperature.

The response surface and contour lines of the interaction of A (extraction time), B (ethanol concentration), C (material-to-liquid ratio), and D (extraction temperature) were shown in Figure 1. The contour plots of various factors can reflect the interaction influence on the response value. Extraction temperature and ethanol concentration had the greatest influence on the extraction yield of TFSR. The contour plots of ethanol concentration and extraction time, ethanol concentration and material-to-liquid ratio, ethanol concentration and extraction temperature were oval, which indicated that the interactions were obvious.

#### 3.1.3. Verification of Predictive Model

The optimal extraction conditions for TFSR were obtained by regression model. The yield of TFSR was 1.08% with the optimal extraction conditions as followed: the ethanol concentration was 57.63%, the material-to-liquid ratio was 1:20.35 (*v*/*w*), the extraction time was 2.57 h, and the extraction temperature was 67.31 °C. For the concentration of ethanol: the changes of extraction yield due to changes of the polarity of the extractant. When the ethanol content was about 60%, the extraction yield of flavonoids reached the highest, and then as the concentration of ethanol increased, the extraction yield of flavonoids decreased. For extraction temperature: the movement of molecules increased with the increased of temperature, so the solubility of flavonoids increased with the increased of temperature, but too high temperature destroyed the structure of heat-sensitive flavonoids [34]. As the extraction time and the material-to-liquid ratio increased, more and more flavonoids dissolved in solvent, and the extraction yield increased and gradually stabilize.

The optimal extraction process was adjusted, according to the actual situations: the ethanol concentration was 58%, the material-to-liquid ratio was 1:20 (*v*/*w*), the extraction time was 2.6 h, and the extraction temperature was 67 °C. With this condition, three repetitive experiments were performed, and the yield of TSFR was highest as 1.04 ± 0.01% (RSD = 0.01%). The optimization extraction yield was very close to the model’s prediction, which meant that this model had good practical significance.

### 3.2. The Flavonoids Contents of the Extracts

It was the first time to systematically investigate the total flavonoids contents of sunflower receptacles. In this study, crude extracts of 58% ethanol were partitioned into four extracts by using different solutions (petroleum ether, ethyl acetate, n-butanol, water) to further enrich the flavonoids contents. As listed in Table 3, it was showed clearly that the EAF had the highest contents of flavonoids, which implied that ethyl acetate was the best extraction solvent to enrich flavonoid compounds from the crude extracts.

### 3.3. Assay of Radical Quenching

The radical scavenging activities of different extract fractions of TFSR were measured in this study. DPPH and ABTS tests are rapid assays that can provide rapid assessment of the radical scavenging ability of phytochemicals in an extract reacting respectively by H-atom transfer and electron transfer with potential antioxidants. Iron ion reducing assay evaluate the reducing ability of an extract which might confirm the presence of potential antioxidants [35,36]. Studies shown that free radical scavenging ability was related to cell aging and inflammation [37,38,39]. Free radical scavenging activities mean that TFSR have potential applications in anti-aging and anti-inflammatory. Therefore, it was necessary to further study the antioxidant activity of the isoquercetin, daidzein and extraction fractions.

#### 3.3.1. DPPH Radical Scavenging Assay

**Figure 2:**
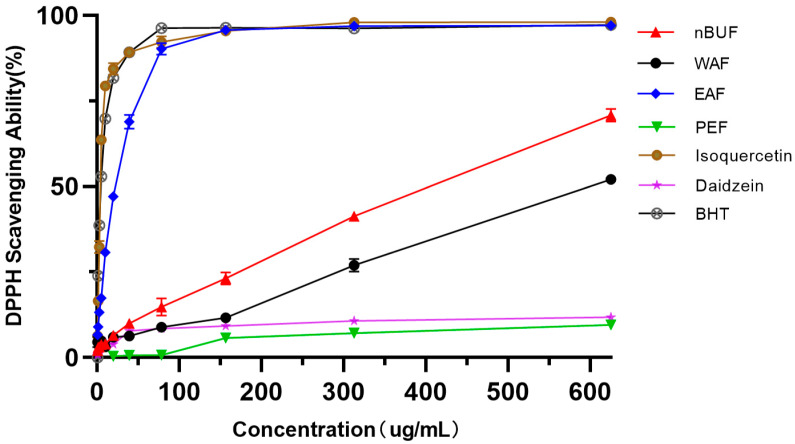
The DPPH scavenging abilities of different preliminary purification fractions and standard samples.

The free radical scavenging activities of the different extraction fractions from sunflower receptacles were determined (Figure 2). The free radical scavenging activities of all samples under the concentration gradient of 0–625 ug/mL were positively correlated and in the order: EAF > nBUF > WAF > PEF. Among all samples, EAF and isoquercetin showed higher free radical scavenging activity. The free-radical scavenging ability of PEF and daidzein were very weak and did not increased as PEF concentration increased.

#### 3.3.2. ABTS Free Radical Scavenging Assay

**Figure 3:**
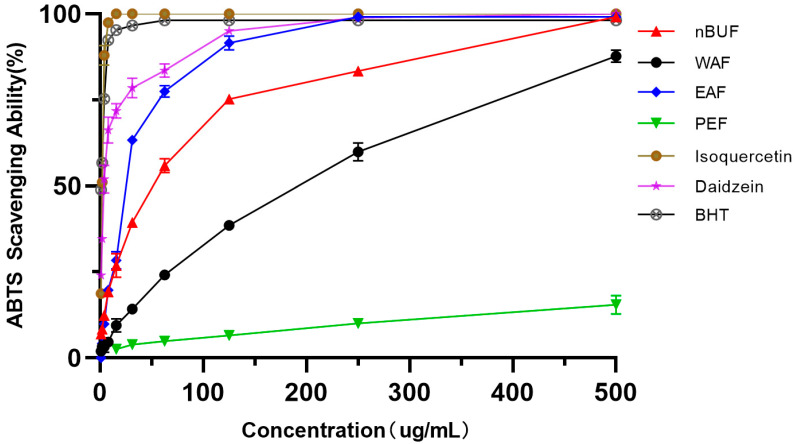
The ABTS scavenging abilities of different preliminary purification fractions and standard samples.

Among the four preliminary purification fractions, EAF had the strongest ABTS scavenging ability, followed by nBuF, WAF and PEF. PEF exhibited the weakest ability in ABTS scavenging ability. The ABTS scavenging ability of isoquercetin and daidzein were stronger than the four preliminary purification fractions. The results were shown in Figure 3.

#### 3.3.3. Iron Ion Reducing Assay

**Figure 4:**
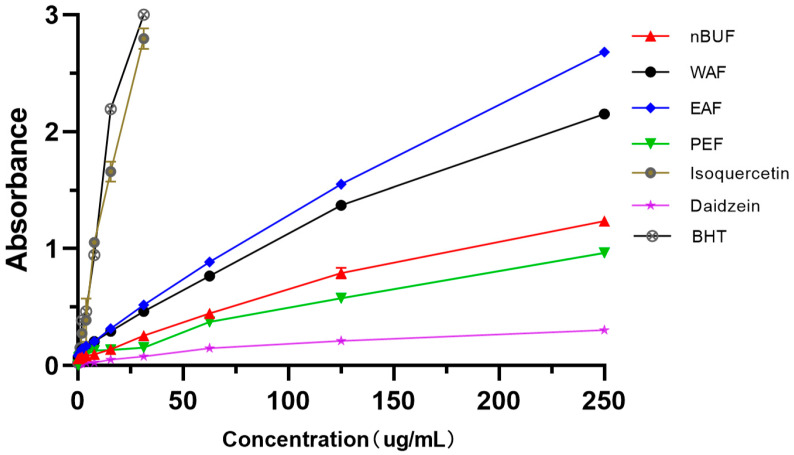
The iron ion reducing abilities of different preliminary purification fractions and standard samples.

Among the four extracts fractions, the iron ion reducing abilities of EAF and WAF were stronger, the reducing ability became stronger as the concentration increased. Among standard samples, BHT and isoquercetin had the higher reduction ability, daidzein had the weakest scavenging ability. (Figure 4).

In summary, EAF had good radical scavenging activities in DPPH and ABTS assays and iron ion reducing activity, EAF also had the highest flavonoids contents in four fractions. Thus, EAF was selected to enrich flavonoids.

### 3.4. Polyamide Resin Preliminary Purification Results

The flavonoids of EAF fraction were further enriched by polyamide resin and eluted with a water–ethanol system. The fractions of A, B, C and D were obtained by elution sequentially with the different concentrations (0%, 30%, 70% and 95%) of ethanol. Fraction B had the highest flavonoids contents as 363.30 ± 2.71 (mg RTE/g DW), which was shown in Table 4. Thus, fraction B was selected to identify the chemical components by UHPLC-HRMS/MS.

### 3.5. UHPLC-HRMS/MS Analysis Results

**Figure 5:**
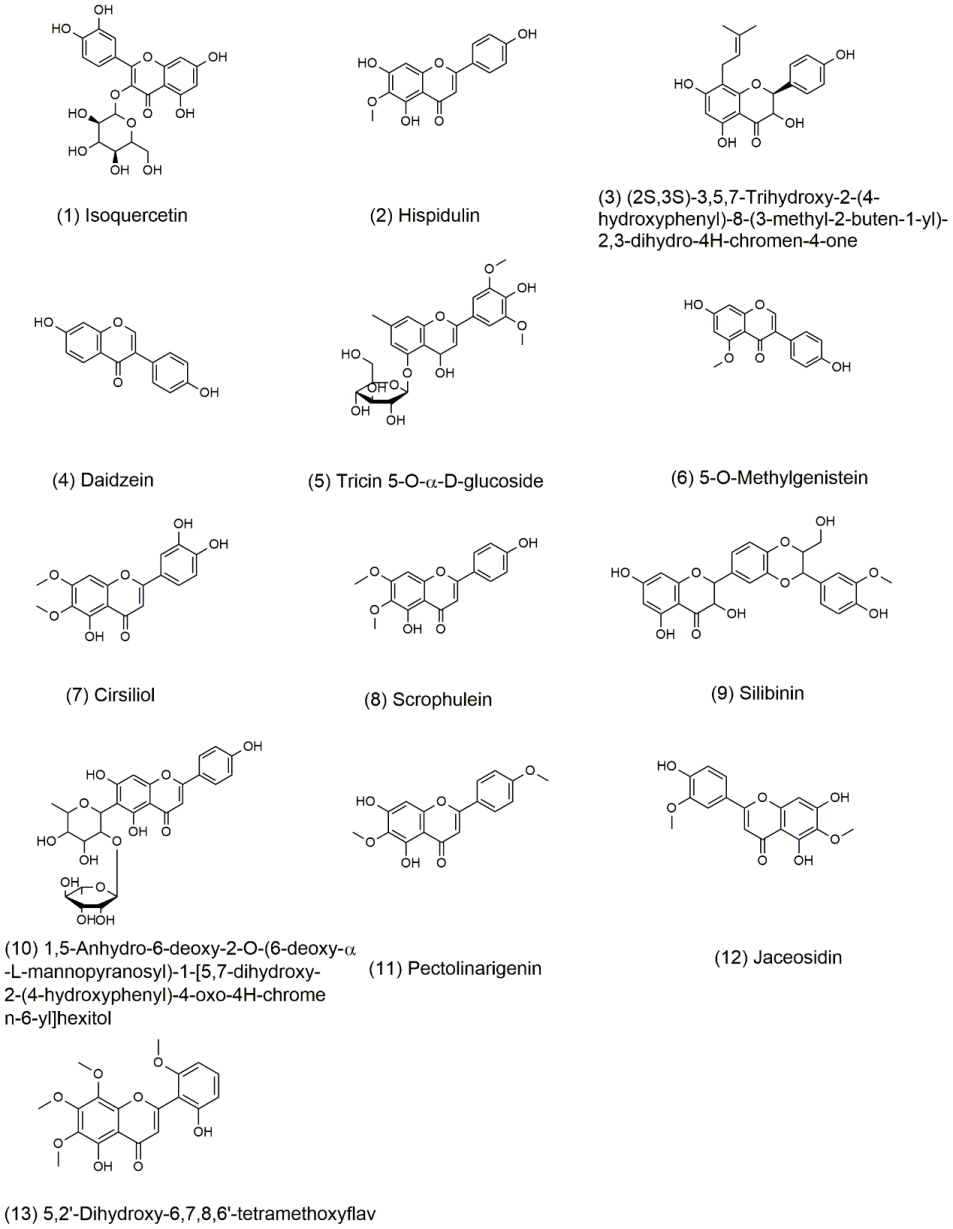
Structure of major flavonoids recognized from fraction B of TFSR by UHPLC-HRMS/MS.

The possible molecular formulas were deduced from high-resolution mass spectrometry information. Thirty-one compounds of flavonoids had best matched in database. The compounds with mass error value of less than 2 ppm were selected and then analyzed by the fragmentation rules of the compounds. Finally, a total of 13 compounds were identified. These compounds have not been reported in sunflower receptacles until now, the retention time and mass spectrum information of the 13 compounds were shown in Table 5. The structure of compounds is shown in Figure 5.

**Figure 6:**
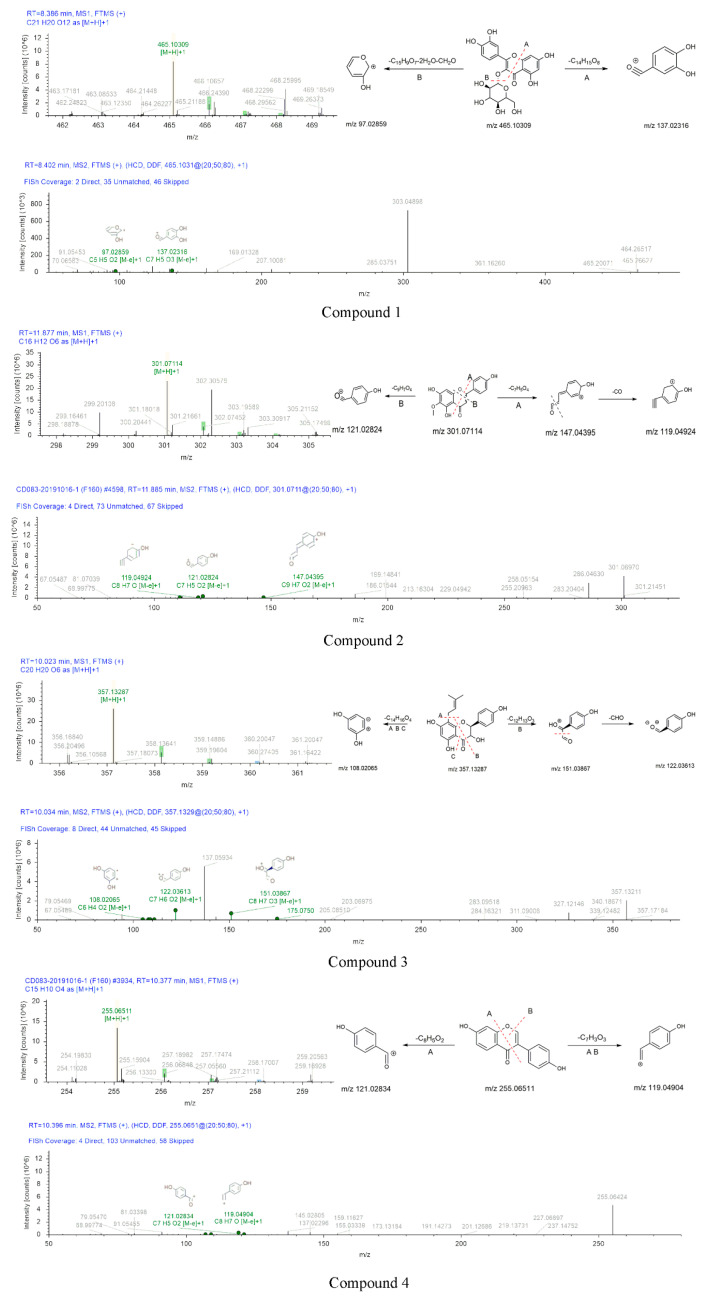

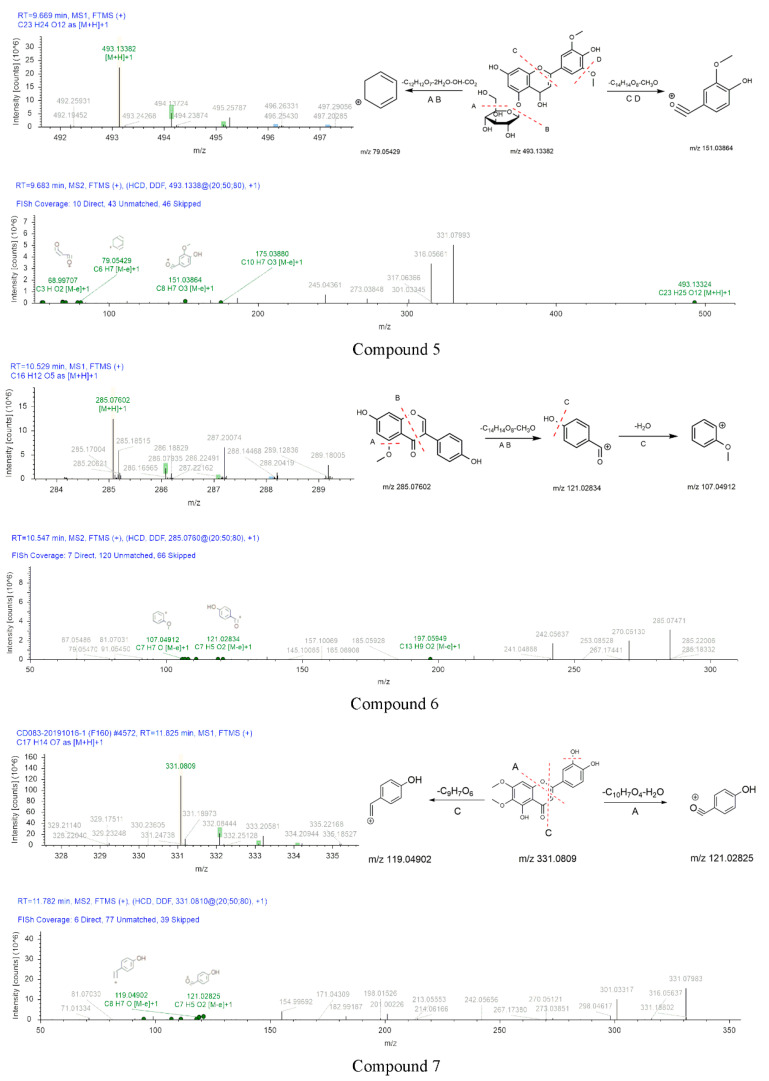
The MS1 and MS2 diagrams of seven compounds and the speculation of the cleavage process.

The mass spectrums of compounds **1**–**7** were exhibited in Figure 6. Compound **1** showed a [M+H]^+^ ion at *m*/*z* 465.10309. This precursor ion generated fragment ions at *m*/*z* 303.04898 (C_15_H_11_O_7_ [M]^+^), 137.02316 (C_7_H_5_O_3_ [M]^+^) and 97.02580 (C_5_H_5_O_2_ [M]^+^). Their formation process was shown in Figure 6. Fragment at *m*/*z* 137 was formed by the breakage of the precursor ion at position A and the losses of C_14_H_15_O_8_. *m*/*z* 303.04898 was formed by the shedding of glucoside in the precursor ion. In the MS2 spectrum, the [M+H]^+^ ion eliminated 301 Da (C_15_H_9_O_7_), 36 Da (2H_2_O), and 30 Da (CH_2_O) to produce *m*/*z* 97.02580. Therefore, compound **1** was identified as isoquercetin. According to the chromatographic conditions in the Appendix A, the existence of isoquercetin was further confirmed. In the chromatogram, the peaks of component B and standard sample appeared at RT = 6.09 and 6.10, and the error of peak time at 0.01 min was reasonable. In the mass spectrum, *m*/*z* 303, *m*/*z* 81 were fragments of standard sample, these fragments also were found in mass spectrum of fraction B. The presence of isoquercetin in fraction B was confirmed by mass spectrum and chromatogram. Details were given in the supplementary material (Figures S1 and S2).

Compound **2** exhibited an [M+H]^+^ ion at *m*/*z* 301.07114 under the positive ionization full scan mode. The information of the fragment ions at *m*/*z* 147.04395 (C_9_H_7_O_2_ [M]^+^), 119.04924 (C_8_H_7_O [M]^+^), 121.02824 (C_7_H_5_O_2_ [M]^+^) were found in MS2. The [M+H]^+^ ion was eliminated 15 Da (CH_3_) to produce *m*/*z* 286.04630. *m*/*z* 147.04395 was formed by the losses of C_7_H_5_O_4_ from the precursor ion. The formation process of *m*/*z* 119.04924 and 121.02824 were shown in Figure 6. The fragment ions of compound **2** were compared with the reported data, the diagnostic ion at *m*/*z* 286.04630, thus, compound **2** was identified as hispidulin [40].

The mother ion of compound **3** was 357.13287 [M+H]^+^_._ The information of fragment ions at *m*/*z* 151.03867 (C_8_H_7_O_3_ [M]^+^), 122.03613 (C_7_H_6_O_2_ [M]^+^), and 108.02065 (C_6_H_4_O_2_ [M]^+^) were detected under the positive spectrum. The formation process was inferred in Figure 6. The fragment ion at *m*/*z* 151.03867 due to the cleavage at B position, the formation of *m*/*z* 122.03613 due to the fragment at *m*/*z* 151.03867 falling 29 Da (CHO), the fragment ion at *m*/*z* 108.02065 was due to the cleavage of A, B and C position. Thus, compound **3** was deduced as (2S,3S)-3,5,7-trihydroxy-2-(4-hydroxyphenyl)-8-(3-methyl-2-buten-1-yl)-2,3-dihydro-4H-chromen-4-one.

Compound **4** was eluted out at 10.377 min and displayed the precursor ion at *m*/*z* 255.06511 (C_15_H_10_O_4_ [M+H]^+^) under positive ionization mode. An amount of secondary MS2 data, such as 119.04904 (C_8_H_7_O [M]^+^) and 121.02834 (C_7_H_5_O_2_ [M]^+^), indicated that the compound **4** may be daidzein based on these results. Compound **4** was temporarily identified.

According to the chromatographic conditions of the Appendix A, the existence of daidzein was further confirmed by compared with standard sample (Supplementary Material). In the chromatogram, the peaks of component B and standard sample both appeared at RT = 6.67. In the mass spectrum, *m*/*z* 137, *m*/*z* 199, *m*/*z* 255 were fully found in the mass spectrum of fraction B and standard sample of daidzein. The presence of daidzein in fraction B was proved by mass spectrum and chromatogram. Details were given in the supplementary material (Figures S3 and S4).

The skeleton structure of flavonoids is C6-C3-C6. The C3 chain of compound **4** was cleaved at position A (Figure 6). Compound **4** (daidzein) belongs to isoflavones, compound **6** (5-O-Methylgenistein) also is an isoflavone. Compound **6** and compound **4** were cleaved at the same position of the C3 chain. The fragments of daidzein provided a basis for the fragment’s analysis of compound **6**. The cross-conjugated system of flavone and the non-cross-conjugated system of isoflavones have an impact on the stability of the C3 chain. The C3 chain of daidzein and isoquercetin were cleaved, the cleavage position has universality and particularity. In addition, the substitution patterns on the C6 rings lead to different types of produce ions and the intensity of relative abundances [41,42].

The peak of compound **5** was observed at 9.669 min in the extracted ion chromatogram. Compound **5** had a quasi-molecular ion [M+H]^+^ at *m*/*z* 493.13382 (C_30_H_48_O_3_). Major fragment ions, such as *m*/*z* 151.03864 (C_8_H_7_O_3_ [M]^+^) and 79.05429 (C_6_H_7_ [M]^+^), were observed in the secondary spectrum. The formation process was presumed as follows: the mother ion was reduced by 310 (C_14_H_14_O_8_) and 31 Da (CH_3_O) compared with the fragment at *m*/*z* 151.03864. [M+H]^+^ ion eliminated 282 (C_12_H_12_O_7_), 36 (2H_2_O), 17 (OH), and 44 Da (CO_2_) to produce *m*/*z* 79.05429. Therefore, compound **5** was preliminarily identified as tricin 5-O-β-D-glucoside.

Compound **6** was eluted on the UPLC system at a retention time of 10.547 min. Its precursor ion [M+H]^+^ (*m*/*z* 285.07602) was detected in MS1. Two major fragment ions at *m*/*z* 121.02834 (C_7_H_5_O_2_ [M]^+^) and 107.04912 (C_7_H_7_O [M]^+^) were revealed in the secondary mass spectrum. Therefore, compound **6** was inferred as 5-O-methylgenistein.

The formation process of compound **7** can be inferred in Figure 6. Compound **7** eluted at 11.825 min had the precursor ion [M+H]^+^ at *m*/*z* 331.0809. The [M+H]^+^ ion eliminated 15 Da (CH_3_) to produce *m*/*z* 316.05637. MS2 fragment at *m*/*z* 119.04902 (C_8_H_7_O [M]^+^) was 211 Da (C_9_H_7_O_6_) less than that of the parent ion. Moreover, diagnostic fragment ions at *m*/*z* 121.02834 were generated by the fragmentation of precursor ion cleaved at position C. The fragment ions of compound **7** were compared with the reported data, the diagnostic ion at *m*/*z* 316.05637 [43]. Thus, the compound **7** was identified as cirsiliol.

Compounds **8**, **9**, **11**, **12** and **13** were detected under positive ionization mode. Compound **10** was identified under negative ionization mode. They may exist in the sunflower receptacles. Based on the mass spectrometry data, we made a preliminary speculation on the compounds of flavonoids from fraction B of EAF.

## 4. Conclusions

This study was the first systematic study on the chemical components and potential radical scavenging activities of TFSR. The optimal extraction yield of TFSR was 1.04% with the ethanol concentration 58%, the material-to-liquid ratio 1:20 (*v*/*w*), the extraction time 2.6 h, and the extraction temperature 67 °C. The crude extracts of TFSR were suspended into water and distribution by increasing polarities (petroleum ether, ethyl acetate, n-butanol), the EAF had the highest total flavonoids contents (191.95 mg RTE/g DW). Polyamide resin had a good enrichment effect on flavonoids in EAF was proved, the flavonoids content of fraction B had increased to 363.30 mg RTE/g DW. Therefore, fraction B was selected for component identification, thirteen chemical compounds of flavonoids were identified by UHPLC-HRMS/MS. The components of isoquercetin and daidzein were identified accurately. In vitro radical scavenging activities analysis showed that EAF and isoquercetin had high potential antioxidant activity. In summary, TFSR as a natural radical scavenger has potential applications in the pharmaceutical industry.

## Figures and Tables

**Table 1:** The design of optimization experimental.

NO.	A Extraction Time (h)	B Ethanol Concentration (%)	C Material to Liquid Ratio (*v*/*w*)	D Extraction Temperature (°C)	Y Extraction Yield (%)
1	2	90	20	85	0.21
2	3	60	20	85	0.90
3	2	60	20	65	1.08
4	2	90	25	65	0.35
5	3	60	15	65	0.93
6	2	60	20	65	1.06
7	2	30	20	45	0.41
8	1	60	25	65	0.71
9	3	30	20	65	0.60
10	2	30	15	65	0.51
11	2	30	25	65	0.59
12	1	60	20	85	0.71
13	2	60	20	65	1.00
14	2	60	20	65	1.00
15	2	60	25	45	0.72
16	2	90	15	65	0.35
17	2	90	20	45	0.34
18	3	60	20	45	0.84
19	2	60	15	85	0.76
20	2	30	20	85	0.62
21	2	60	20	65	1.07
22	3	60	25	65	0.94
23	1	30	20	65	0.55
24	1	60	15	65	0.70
25	2	60	25	85	0.77
26	2	60	15	45	0.51
27	3	90	20	65	0.56
28	1	60	20	45	0.64
29	1	90	20	65	0.22

**Table 2:** Results of response surface regression models.

Source	Sum of Squares	Df	Mean-Square	F-Value	*p*-Value	Significance
Model	1.800	14	12.86	79.78	<0.0001	* * *
A	0.127	1	12.65	78.47	<0.0001	* * *
B	0.130	1	13.01	80.67	<0.0001	* * *
C	0.008	1	0.79	4.93	0.0434	*
D	0.022	1	2.20	13.67	0.0024	* *
AB	0.022	1	2.20	13.62	0.0024	* *
AC	0.022	1	0.00	0.01	0.9172	
AD	0.000	1	0.00	0.02	0.8785	
BC	0.000	1	0.14	0.85	0.3712	
BD	0.001	1	2.85	17.70	0.0009	* * *
CD	0.010	1	1.01	6.27	0.0253	*
A^2^	0.046	1	4.61	28.61	0.0001	* * *
B^2^	1.360	1	136.26	845.13	<0.0001	* * *
C^2^	0.131	1	13.15	81.55	<0.0001	* * *
D^2^	0.239	1	23.97	148.63	<0.0001	* * *
Residual	0.022	14	0.1612			
Lack of Fit	0.172	10	0.1718	1.28	0.4390	
Pure Error	0.005	4	0.1347			
Cor Total	1.820	28				
*R*^2^ = 0.9876						
*R*^2^_Adj_ = 0.9752						
CV = 5.93						

* * * indicates that the difference is extremely significant (*p* < 0.001). * * indicates that the difference is highly significant (*p* < 0.01). * indicates that the difference is significant (*p* < 0.05). df indicates that degree of freedom.

**Table 3:** The Flavonoids Contents of the Different Fractions.

Fraction	Contents of Flavonoids (mg RTE/g DW)
PEF	15. 14 ± 0.29 ^d^
EAF	191.95 ± 2.94 ^a^
nBUF	73.41 ± 2.04 ^b^
WAF	46.46 ± 0.56 ^c^

PEF: petroleum ether fraction, EAF: ethyl acetate fraction, nBUF: n-butanol fraction, WAF: water fraction. a, b, c and d represent a significant difference (*p* < 0.05). Values are the mean ± standard deviation of three independent experiments.

**Table 4:** The flavonoids contents of the eluted fraction A, B, C and D.

Fraction	Ethanol Elution Concentration	Contents of Flavonoids (mg RTE/g DW)
A	0%	210.53 ± 3.01 ^a^
B	30%	363.30 ± 2.71 ^b^
C	70%	174.70 ± 2.52 ^c^
D	95%	46.96 ± 1.19 ^d^

a, b, c and d represent significant differences (*p* < 0.05). Values are the mean ± standard deviation of three independent experiments.

**Table 5:** UHPLC-HRMS/MS data of flavonoids in TFSR.

No	Full Name	Ion Mode	Predicted Formula	Rt (min)	Measured (*m*/*z*)	Theoretical (*m*/*z*)	Error (ppm)	Fr (*m*/*z*)
1	Isoquercetin	+	C_21_H_20_O_12_	8.36	464.09580	464.09548	−0.634	465.10309 303.04990 137.02316 97.02859
2	Hispidulin	+	C_16_H_12_O_6_	11.87	300.06390	300.06339	−1.572	301.07114 147.04395 286.04630 121.02824 119.04924
3	(2S,3S)-3,5,7-Trihydroxy-2-(4-hydroxyphenyl)-8-(3-methyl-2-buten-1-yl)-2,3-dihydro-4H-chromen-4-one	+	C_20_H_20_O_6_	10.02	356.12560	356.12599	1.099	357.13287 175.0750 151.03867 122.03613 108.02065
4	Daidzein	+	C_15_H_10_O_4_	10.36	254.05780	254.05791	0.300	255.06511 137.02350 119.04904 121.02834
5	Tricin 5-O-β-D-glucoside	+	C_23_H_24_O_12_	9.69	492.12620	492.12678	1.156	493.13382 175.03880 151.03864 79.05429
6	5-O-Methylgenistein	+	C_16_H_12_O_5_	10.54	284.06870	284.06847	−0.948	285.07602 197.05949 121.02834 107.04912
7	Cirsiliol	+	C_17_H_14_O_7_	11.81	330.07370	330.07395	0.788	331.08093 316.05637 121.02825 119.04902
8	Scrophulein	+	C_17_H_14_O_6_	10.53	314.07940	314.07904	−1.131	315.08667 297.08643
9	Silibinin	+	C_25_H_22_O_10_	10.06	482.12210	482.12130	−1.766	483.12943 163.03848 147.04361
10	1,5-Anhydro-6-deoxy-2-O-(6-deoxy-α-L-mannopyranosyl)-1-[5,7-dihydroxy-2-(4-hydroxyphenyl)-4-oxo-4H-chromen-6-yl] hexitol	-	C_27_H_30_O_13_	10.4	562.16870	562.16864	−0.131	561.16162 339.08701
11	Pectolinarigenin	+	C_17_H_14_O_6_	12.29	314.07940	314.07904	−1.131	315.08682 161.09552
12	Jaceosidin	+	C_17_H_14_O_7_	27.98	330.07460	330.07395	−1.875	331.19049 316.05704
13	5,2’-Dihydroxy-6,7,8,6’-tetramethoxyflave	+	C_19_H_18_O_8_	13.75	374.11050	374.10017	−0.825	375.10776 345.05911 169.01268

Rt: retention time; Fr: fragment.

## Data Availability

Data is contained within the article or supplementary material.

## Supplementary Materials

The following are available online, Figure S1. The chromatogram of fraction B (a) and isoquercetin standard sample (b). Figure S2. The mass spectrum of fraction B (a) and isoquercetin standard sample (b). Figure S3. The chromatogram of fraction B (a) and daidzein standard sample (b). Figure S4. The mass spectrum of fraction B (a) and daidzein standard sample (b). Figure S5. The MS1 diagrams of six compounds.

## Abbreviations

TFSR	Total flavonoids in sunflower receptacles
UHPLC-HRMS/MS	Ultimate 3000 Nano LC System coupled to a Q Exactive HF benchtop Orbitrap mass spectrometer
DPPH	2,2-Diphenyl-1-picrylhydrazyl
ABTS	2,2-Azino-bis (3-ethylbenzothiazoline-6-sulfonic acid)
BHT	Butylated hydroxytoluene
PEF	Petroleum ether fraction
EAF	Ethyl acetate fraction
nBUF	n-Butanol fraction
WAF	Water fraction
Df	Degree of freedom
RT	Retention time
Rt	Retention time
Fr	Fragment
CUPRAC	Cupric ion reducing antioxidant capacity
ANOVA	One-way analysis of variance

## Appendix A. Details of Comparison with Standards

### Appendix A.1. Chromatographic Conditions

RP-C18 column (150 × 210 mm, 1.8 μm; Welch) was used. The mobile phase was 0.1% formic acid aqueous solution (A) to 0.1% formic acid acetonitrile (B). Gradient elution proceeded as follows: 0–0.5 min, 98% A; 0.5–6.5 min, 98% A → 2% A; 6.5–9 min, 2% A → 2% A; 9–9.3 min, 2% A → 98% A; 9.3–10 min, 98% A → 98% A). The flow rate was 0.3 mL/min. Autosampler temperature was 10.0 °C, column temperature was 35 °C; injection needle height was 2.00 mm, and injection volume was 10 µL.

### Appendix A.2. Mass Spectrometry Conditions

Ion source was ESI under positive and negative ion switching scanning mode. Scanning range was *m*/*z* 100–1500. The detection method used was parallel reaction monitoring. The resolution was 70,000 in full mass and 17,500 in dd-MS2; The ion spray needle voltage was 3.8 kV (positive), capillary temperature was 300 °C, collision gas was high-purity argon (purity ≥ 99.999%), sheath gas was nitrogen (purity ≥ 99.999%) and 40Arb, auxillary gas was nitrogen (purity ≥ 99.999%, heater temp, 350 °C, and data collection time was 30.0 min.
