# Supplementary material for: Extraction, Radical Scavenging Activities, and Chemical Composition Identification of Flavonoids from Sunflower (Helianthus annuus L.) Receptacles
Source: Molecules. 2021 Jan 14;26(2):403. doi: 10.3390/molecules26020403 (PMC7828773; doi:10.3390/molecules26020403)
Supplement: Supplementary file 1 [file molecules-26-00403-s001.pdf]

Supplementary Material

Extraction, Radical Scavenging Activities, and Chemical Composition Identification of Flavonoids from Sunflower (*Helianthus annuus* L.) Receptacles

Zian Qiao [1,2,‡], Lu Han [1,3,‡], Xinsheng Liu [1,3], Huining Dai [1], Changmin Liu [1], Min Yan [1], Wannan Li [1], Weiwei Han [1,4], XinLu Li [1], Silu Huang [1] and Bo Gao [1,2,*]

[1] School of Life Sciences, Jilin University, Changchun 130012, China; qiaoza18@mails.jlu.edu.cn (Z.-A.Q.); luhan@jlu.edu.cn (L.H.); liuxs18@mails.jlu.edu.cn (X.-S.L.); daihn18@mails.jlu.edu.cn (H.-N.D.); liucz19@mails.jlu.edu.cn (C.-M.L.); xinlu19@mails.jlu.edu.cn (X.-L.L.); liwannan@jlu.edu.cn (W.-N.L.); yanmin19@mails.jlu.edu.cn (M.Y.); slhuang18@mails.jlu.edu.cn (S.-L.H.); weiweihan@jlu.edu.cn (W.-W.H.);

[2] Third-Level Laboratory of National Administration of Traditional Chinese Medicine, Jilin University, Changchun 130012, China

[3] Key Laboratory for Evolution of Past Life and Environment in Northeast Asia, Jilin University, Ministry of Education, Changchun 130012, China

[4] Key Laboratory for Molecular Enzymology and Engineering, Jilin University, Ministry of Education, Changchun 130012, China

* Correspondence: gaobo@jlu.edu.cn; Tel.: +86-131-3443-5290; Fax: +86-431-85155127

‡ These authors contributed equally to this work.

Table of contents

Figure A1. The chromatogram of fraction B (**a**) and isoquercetin standard sample (**b**).

Figure A2. The mass spectrum of fraction B (**a**) and isoquercetin standard sample (**b**).

Figure A3. The chromatogram of fraction B (**a**) and daidzein standard sample (**b**).

Figure A4. The mass spectrum of fraction B (**a**) and daidzein standard sample (**b**).

Figure A5. The MS1 diagrams of six compounds.

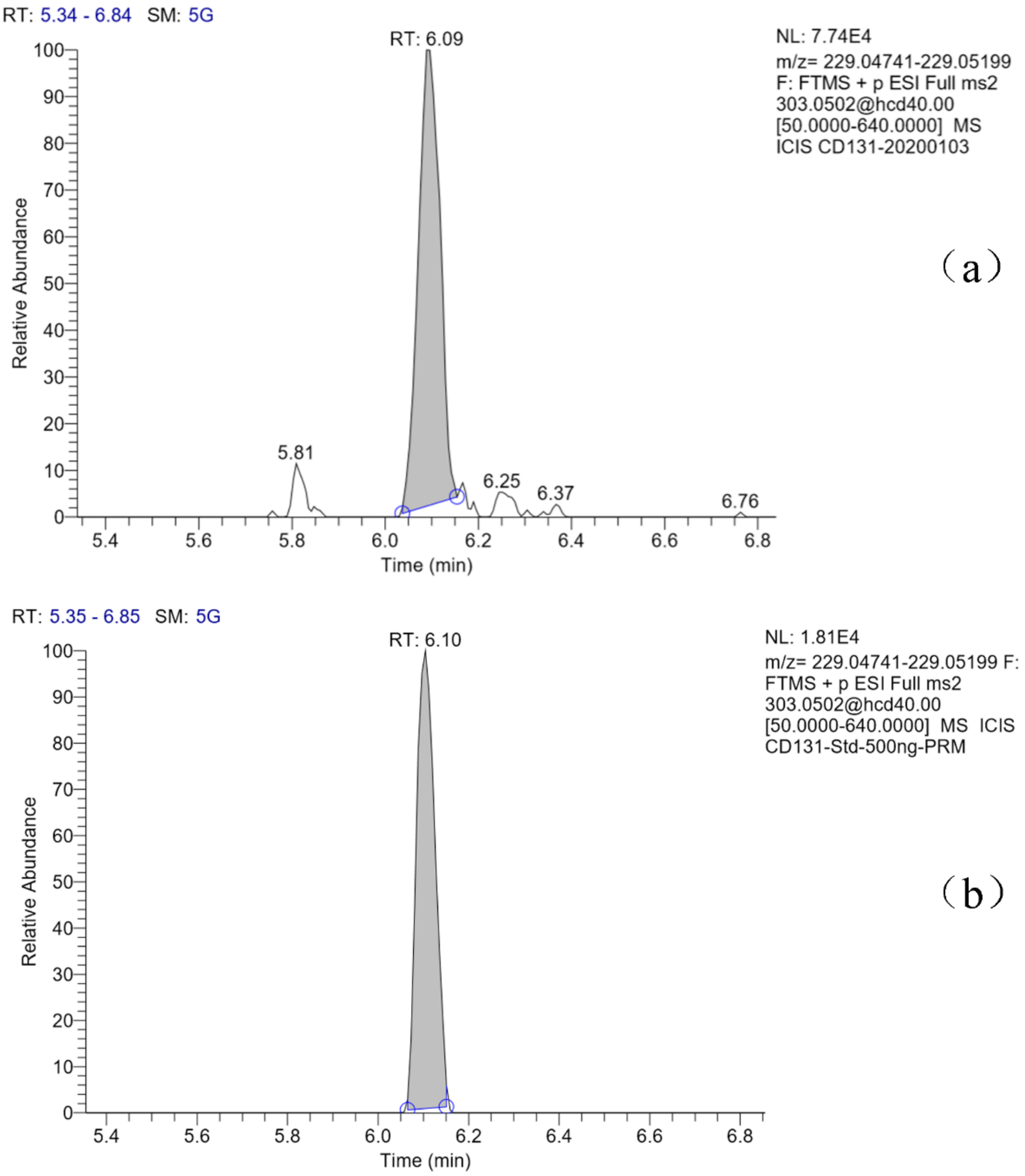

Figure A1. The chromatogram of fraction B (**a**) and isoquercetin standard sample (**b**).

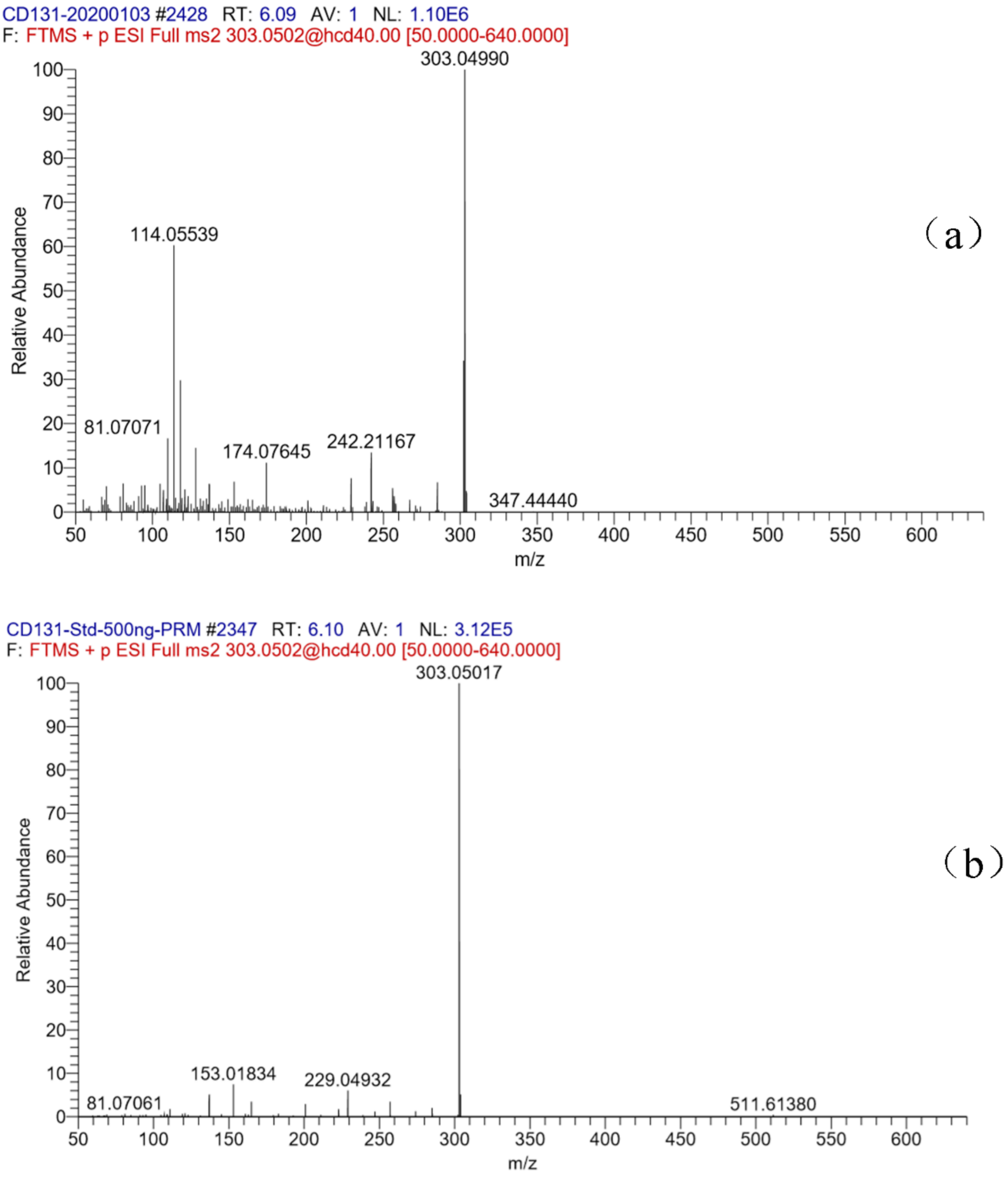

Figure A2. The mass spectrum of fraction B (**a**) and isoquercetin standard sample (**b**).

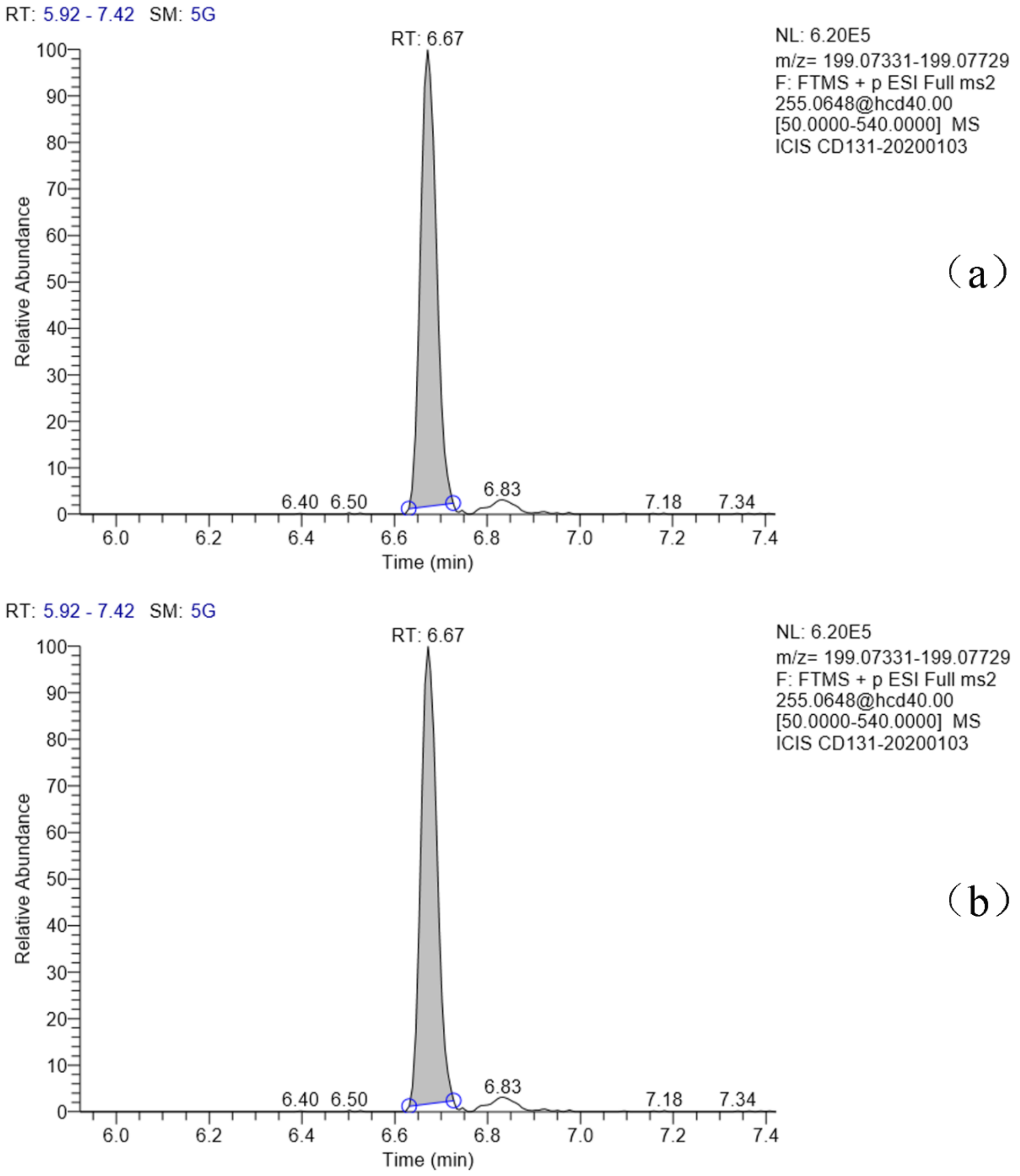

Figure A3. The chromatogram of fraction B (**a**) and daidzein standard sample (**b**).

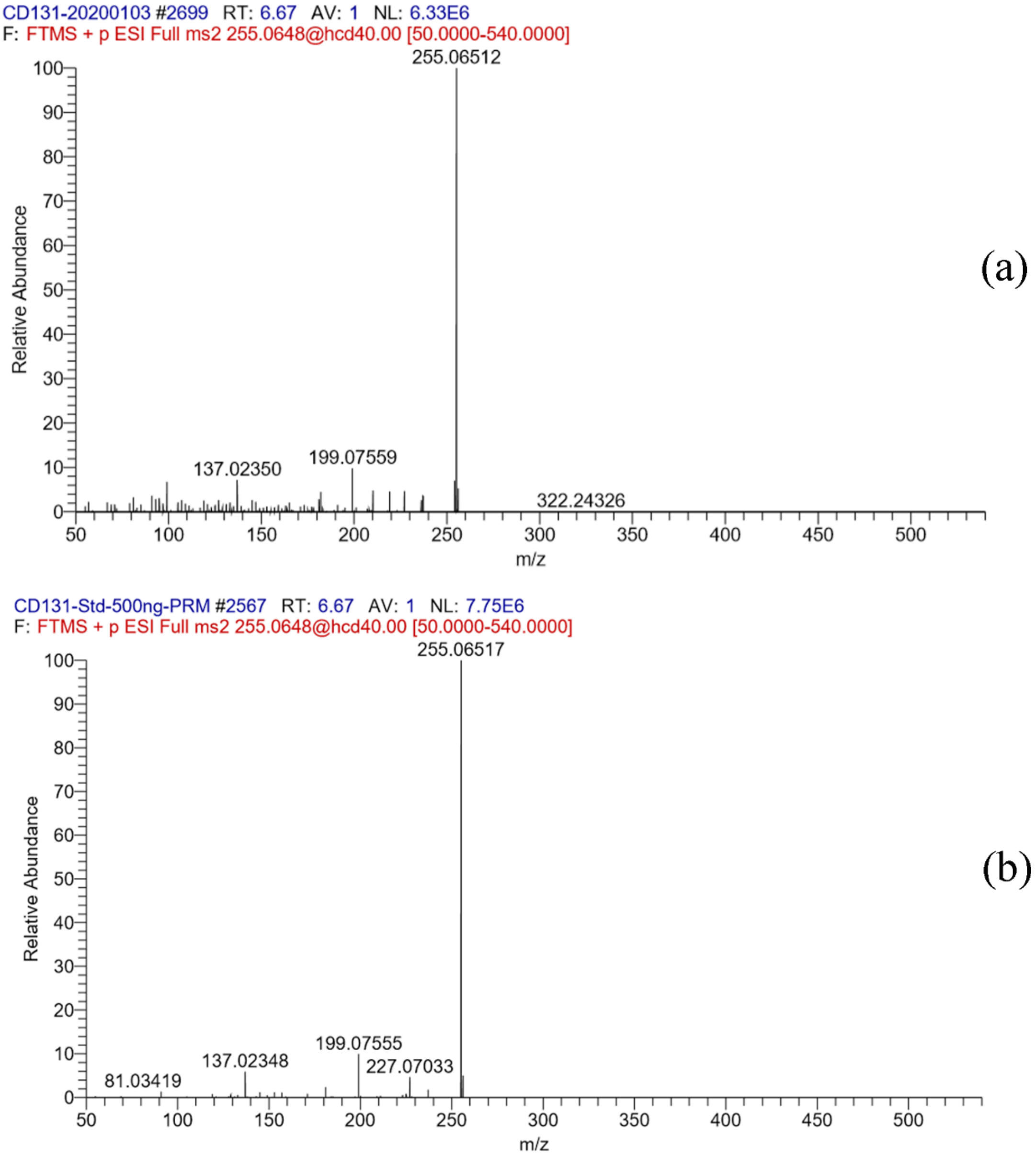

Figure A4. The mass spectrum of fraction B (**a**) and daidzein standard sample (**b**).

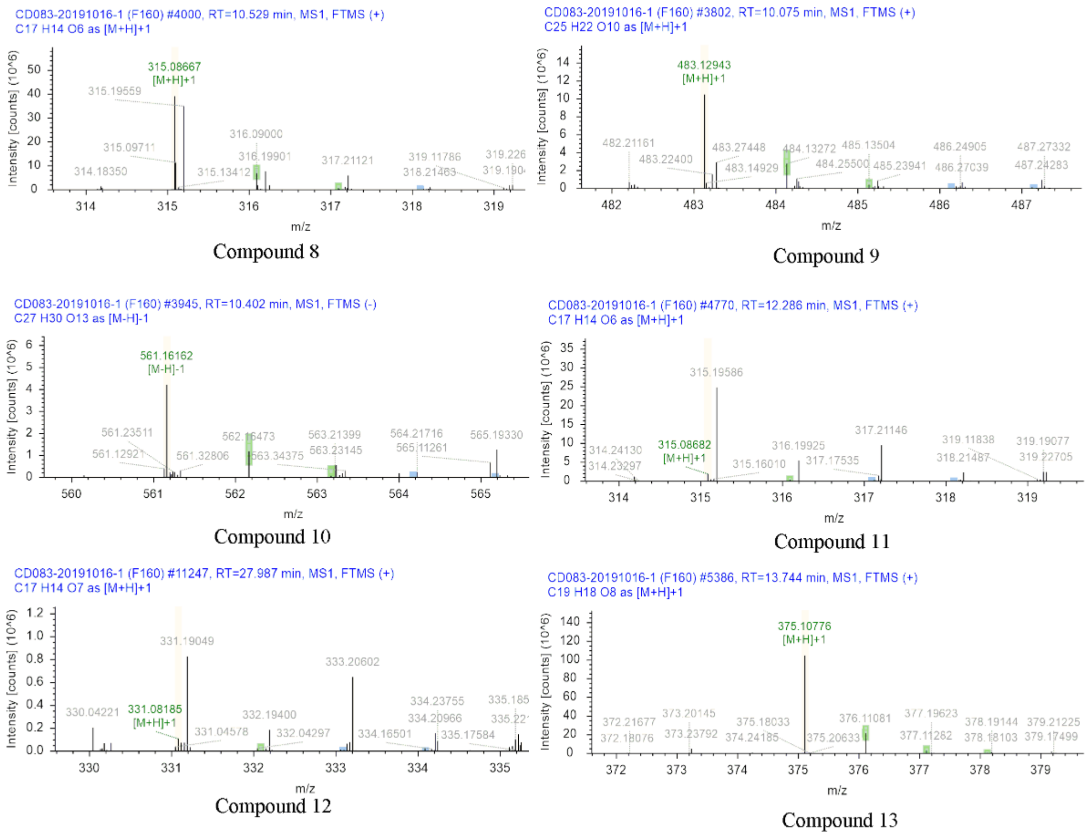

Figure A5. The MS1 diagrams of six compounds.
